# Prioritizing Long‐Term Care Services for Disabled Older Adults: A Cross‐Sectional Study Using the Kano Model and Entropy‐Weighted TOPSIS

**DOI:** 10.1155/jonm/2094897

**Published:** 2026-08-12

**Authors:** Li Chen, Liu Yang, Jialin Wang, Ying Xia, Jiling Ye, Ying Huang, Qi An

**Affiliations:** ^1^ College of Nursing, Chengdu University of Traditional Chinese Medicine, Chengdu 610075, China, cdutcm.edu.cn; ^2^ Department of Oncology, Western Theater Command General Hospital, Chengdu 610083, China; ^3^ Palliative Care Center, Chengdu Eighth People’s Hospital (Geriatric Hospital of Chengdu Medical College), Chengdu 610083, China

**Keywords:** disabled older adults, Kano model, long-term care needs, supply priorities, TOPSIS analysis

## Abstract

**Background:**

Understanding the hierarchical structure and supply priorities of long‐term care (LTC) needs among disabled older adults is essential for responsive care systems. However, existing assessments fail to translate findings into actionable resource allocation strategies, leaving nursing managers without evidence‐based guidance for prioritization.

**Aim:**

To identify the hierarchical structure of LTC service demands among disabled older adults using the Kano model and to establish an evidence‐based supply priority order through integrated entropy‐weighted TOPSIS analysis, thereby providing nursing managers with a quantifiable sequence for phased resource allocation.

**Methods:**

A cross‐sectional survey was conducted from January to November 2024 among 585 disabled older adults recruited from six nursing institutions and six communities across three districts of Chengdu. Participants completed a self‐designed Kano model questionnaire covering 52 service items. Kano analysis was used to classify service attributes into must‐be, one‐dimensional, attractive, and indifferent categories, and entropy‐weighted TOPSIS was subsequently applied to determine supply priorities based on relative closeness (*C*_*i*_) values.

**Results:**

Among the 52 items, Kano analysis identified 12 (23.1%) as must‐be, 14 (26.9%) as one‐dimensional, 10 (19.2%) as attractive, and 16 (30.8%) as indifferent. *C*_*i*_ values ranged from 0.007 to 0.905. Within one‐dimensional attributes, vital signs monitoring (*C*_*i*_ = 0.905) and oral medication administration (*C*_*i*_ = 0.890) ranked highest. Among attractive attributes, cognitive training had the highest priority (*C*_*i*_ = 0.615). Notably, 11 of 15 traditional Chinese medicine (TCM) nursing services (73.3%) fell into attractive or one‐dimensional categories.

**Conclusion:**

The integrated Kano–TOPSIS framework identifies LTC need hierarchies and supply priorities among disabled older adults. Must‐be services need basic guarantees; one‐dimensional services drive quality; and attractive services, especially TCM nursing and cognitive training, boost satisfaction. This user‐centered approach guides phased resource allocation and responsive LTC design.

**Implications for Nursing Management:**

The findings support a tiered resource allocation strategy based on the Kano–TOPSIS framework. Must‐be services should be guaranteed as a basic care package. One‐dimensional services should be prioritized for quality monitoring and performance evaluation. Attractive services can be developed as value‐added modules to enhance cultural responsiveness. A phased approach is recommended: must‐be first, then one‐dimensional, and finally attractive according to local capacity. These measures enable nursing managers to translate needs assessment into actionable allocation decisions and bridge the supply–demand gap.

## 1. Introduction

Long‐term care (LTC) refers to ongoing support services provided to individuals who have lost intrinsic capacity due to illness or disability [1]. With the acceleration of global population aging, older adults have become the largest user group of LTC. In China, the population aged 60 years and above reached 264 million by the end of 2020, accounting for 18.7% of the total population [2], and is projected to exceed 400 million by 2035 [3]. Within this large population, there is a subgroup of older adults who have lost partial or complete self‐care ability due to physical or cognitive impairments—referred to as “disabled older adults.” Currently, approximately 45 million disabled or semidisabled older adults reside in China, accounting for 17.1% of the elderly population; this number is projected to reach 61.68 million by 2030 and 97.5 million by 2050 [4]. Most of these individuals suffer from multiple chronic conditions, resulting in an exceptionally high demand for continuous care and support [5, 6]. In short, disabled older adults constitute the largest and most rapidly growing user group of LTC, and their needs have become an urgent policy priority.

This high demand, however, is met with inadequate supply. At the family level, traditional care models are weakening due to rapid urbanization, declining fertility rates, and the growing prevalence of “empty‐nest” households [7], placing the care burden on a limited number of family members—a phenomenon often described as “one person disabled, the whole family disrupted” [8]. Family caregivers frequently experience high levels of stress, with increased risks of anxiety, depression, and social isolation [9]. At the societal level, institutional care resources are unevenly distributed and often homogeneous, while community‐based home care systems remain underdeveloped and ill‐equipped to deliver the specialized, personalized care that disabled older adults require [10, 11]. In response, the Chinese government has introduced policy initiatives such as the 2016 pilot LTC Insurance program [7, 12]. However, policy frameworks alone cannot bridge the gap; the effectiveness of these efforts ultimately depends on accurately assessing what care is truly needed. Unfortunately, current needs assessments often rely on simplistic checklists or provider‐defined categories that fail to capture the hierarchical and nuanced nature of user priorities. As a result, high rates of unmet care needs persist among this population [13, 14], largely because a systematic understanding of “true need” remains lacking.

Despite its recognized value in geriatric and chronic care, traditional Chinese medicine (TCM) nursing has been largely excluded from LTC needs assessments. Grounded in holistic philosophy, TCM nursing emphasizes the balance of yin and yang, the smooth flow of qi and blood through meridians, and the principles of “preventive treatment” and “treatment based on syndrome differentiation”—distinguishing it from conventional Western nursing [15]. Its nonpharmacological modalities, such as moxibustion, acupoint application, herbal soaking, and cupping, are particularly suitable for disabled older adults with multimorbidity and functional decline. Yet this omission has left a critical gap in understanding the full spectrum of care needs among this population [16]. Moreover, most existing studies rely on descriptive statistics or conventional regression analyses, which cannot differentiate essential services from expected or value‐added ones [17, 18]. Beyond these limitations, a persistent disconnect exists between needs assessment and service provision: research findings are rarely translated into actionable supply priorities or tiered resource allocation strategies, leading to inefficient resource deployment and a chronic misalignment between supply and actual demands [19, 20].

These limitations highlight the need for a more rigorous analytical approach. This study introduces the Kano model—a user‐centered framework that classifies service requirements into five categories (must‐be, one‐dimensional, attractive, indifferent, and reverse) based on their impact on satisfaction [21, 22]. To overcome its inability to prioritize within the same category, we further integrate the Kano model with entropy‐weighted Technique for Order Preference by Similarity to an Ideal Solution (TOPSIS), which calculates the relative closeness coefficient (*C*_*i*_) of each service to determine supply urgency [23]. This combined approach enables evidence‐based, sequential resource allocation, ensuring that the most urgent needs are addressed first. Specifically, this study aims to (1) identify the hierarchical structure of LTC service demands among disabled older adults using the Kano model; (2) establish a tiered supply priority using entropy‐weighted TOPSIS; and (3) explore the potential role of TCM nursing services as value‐added components in a culturally responsive LTC system. By translating needs assessment into actionable management strategies, this framework seeks to bridge the persistent gap between supply and demand in nursing care.

## 2. Methods

### 2.1. Study Design

A cross‐sectional study was conducted from January to November 2024 in three administrative districts of Chengdu City, Sichuan Province, China.

### 2.2. Data Source and Collection

#### 2.2.1. Sampling Method

A multistage stratified convenience sampling method was employed to recruit participants. In the first stage, to account for the marked heterogeneity in care resources, healthcare infrastructure, and socioeconomic conditions across Chengdu, we randomly selected one administrative district from each of three geographically distinct zones: the central urban area, the suburban area, and the peri‐urban area. In the second stage, two nursing institutions and two communities were conveniently selected from each district, yielding a total of six nursing institutions and six communities as survey sites.

#### 2.2.2. Participants

The inclusion criteria were as follows: (1) aged 60 years or older; (2) classified as disabled according to the national standard “Specification for Elderly Capacity Assessment” (GB/T 42195‐2022); (3) able to communicate and understand the questionnaire content, either independently or with assistance from their primary caregiver; and (4) provided written informed consent.

Exclusion criteria were as follows: (1) diagnosed with dementia, Parkinson’s disease, or other severe mental illnesses affecting communication; and (2) in the acute phase of illness or had severe cardiac, pulmonary, or renal dysfunction.

#### 2.2.3. Sample Size

As per Kendall’s [24] sample size estimation formula for descriptive studies: *N* = *n* × (5–10) × (1 + 10%), and Hulland et al. [25] stipulated the criterion for Kano model questionnaires as *N* ≥ *n* × 10 with a minimum of 200 respondents. This study’s questionnaire contained 52 items; we conservatively used the multiplier of 10 to calculate a required minimum sample size of *N* = 52 × 10 × (1+10%) = 572, which also met Hulland et al.′s threshold of *N* ≥ 520. Combining both criteria, we set the target sample at 572 participants. A total of 610 questionnaires were distributed, yielding 585 valid responses with a 95.9% valid rate exceeding the minimum requirement. “*N*” denotes sample size, and “*n*” refers to the number of questionnaire items.

### 2.3. Research Instruments

#### 2.3.1. General Information Questionnaire

A self‐developed questionnaire was used to collect participants′ sociodemographic and health‐related characteristics, including gender, age, living arrangement, marital status, educational level, number of children, primary caregiver, monthly household income per capita, main source of income, type of medical insurance, number of chronic diseases, and number of medications taken.

#### 2.3.2. Elderly Ability Assessment

Disability status was evaluated using the national standard “Specification for Elderly Capacity Assessment” (GB/T 42195‐2022) issued by the Ministry of Civil Affairs of China [26]. This assessment tool comprises four dimensions: self‐care ability (8 items), basic motor ability (4 items), mental status (9 items), and sensory perception and social engagement (5 items), with a total of 26 items. Each item is scored, and the total score determines the level of disability. Based on the total scores, participants were classified into four categories: mild disability (scores 66–89), moderate disability (scores 46–65), severe disability (scores 30–45), and complete disability (scores 0–29).

#### 2.3.3. Kano Model Questionnaire on Long‐Term Care Service Needs

##### 2.3.3.1. Questionnaire Development

The Kano model questionnaire was developed by integrating multiple sources. The primary framework was the national “List of Recommended Nursing Service Projects,” jointly issued by the National Health Commission, the China Banking and Insurance Regulatory Commission, and the National Administration of TCM. This document organizes nursing services into four dimensions: Daily Care, Medical Care and Rehabilitation, Psychological Care, and TCM Nursing. Given its official endorsement, comprehensive coverage, and scientific classification, we adopted this four‐dimensional structure as the initial framework. This was supplemented by a comprehensive literature review on LTC needs and Kano model applications, as well as insights drawn from our research team’s previous project, “Elderly Ability Assessment and Care Service Indicators in Chengdu Nursing Institutions.”

An expert panel comprising eight specialists in geriatric nursing, community nursing, rehabilitation nursing, and TCM nursing was convened to review and refine the initial item pool. All experts held senior professional titles (professor or chief/associate chief superintendent nurse) and had extensive clinical and academic experience in their respective fields, with a mean of over 24 years of practice (see Table 1). We conducted one round of expert review through a structured symposium, during which the experts were asked to evaluate each item for relevance, clarity, clinical applicability, and cultural appropriateness. Based on their collective feedback, the following modifications were made: A2 “hair cleaning” was merged into A1 “head and facial cleansing and grooming”; “warm water sponge bath” and “showering” were merged into A5 “assistance with sponge bath and showering”; “assistance with defecation and guidance” and “incontinence care and guidance” were merged into A8 “excretion care and guidance”; “oral medication administration” and “medication guidance” were merged into B10 “oral medication administration and guidance”; “indwelling gastric tube care” and “indwelling urinary catheter care” were merged into B13 “indwelling catheter care”; “anal tube” and “enema” were merged into B14 “enema and anal tube care”; “local medication administration” and “rectal medication administration” were merged into B20 “medication administration guidance and care”; “physical health assessment and post‐assessment education” and “health education” were merged into B22 “physical health assessment and education”; and several training items were consolidated into B23 “physical function rehabilitation training.” Items such as perineal care, assistance with effective coughing, and assistance with hot water bottle use for warmth, as well as CVC maintenance, PICC maintenance, and infusion port care, were deleted. The three items in the Psychological Care dimension were deemed appropriate and retained without modification. Through this process, the initial 73‐item list was refined to 52 items, resulting in the final Kano Questionnaire on LTC Needs of the Disabled Elderly, comprising four dimensions and 52 items. Subsequently, a pilot test was conducted with 20 disabled older adults and their caregivers to evaluate comprehensibility and acceptability, which informed further cultural and linguistic refinements.

**TABLE 1:** Composition and qualifications of the expert panel.

Expert	Academic degree	Professional title	Specialty	Years of experience
A	Master	Professor	Geriatric nursing	21
B	Master	Associate Professor	Community nursing	16
C	Master	Chief Superintendent Nurse	Geriatric nursing	19
D	Doctor	Professor	Community nursing	34
E	Master	Chief Superintendent Nurse, Professor	Community nursing	31
F	Master	Superintendent Nurse	Rehabilitation nursing	14
G	Bachelor	Associate Chief Superintendent Nurse	TCM nursing	31
H	Master	Chief Superintendent Nurse	TCM nursing	28

##### 2.3.3.2. Questionnaire Structure

The final questionnaire comprised 52 items organized into four dimensions: Daily Care (13 items), Medical Care and Rehabilitation (27 items), Psychological Care (3 items), and TCM Nursing (9 items). The detailed service items within each dimension are presented in Table 2. Consistent with the standard Kano model format, each service item was evaluated using two complementary questions: a functional question (“How would you feel if this service were provided?”) and a dysfunctional question (“How would you feel if this service were NOT provided?”). For each question, respondents selected one of five response options: “I like it,” “I expect it,” “Neutral,” “I can tolerate it,” or “I dislike it.” The responses were then classified according to the standard Kano evaluation matrix (Table 3). According to the Kano model, each service item is classified into one of five categories based on respondents′ answers to the paired questions. Must‐be attributes are basic services whose absence causes strong dissatisfaction, although their presence does not substantially increase satisfaction. One‐dimensional attributes have a linear relationship with satisfaction; better provision increases satisfaction, whereas poor provision causes dissatisfaction. Attractive attributes are value‐added services that substantially increase satisfaction when provided but do not cause dissatisfaction when absent. Indifferent attributes have little influence on satisfaction regardless of whether they are provided. Reverse attributes indicate that provision of the service may actually reduce satisfaction. This paired questioning format, comprising a functional and a dysfunctional question for each service item, follows the standard Kano questionnaire design [27].

**TABLE 2:** Kano questionnaire on long‐term care service needs for disabled older adults.

Dimension	Code	Description of service items
Daily Care	A1	Head, face cleaning and grooming
	A2	Oral cleaning
	A3	Hand and foot cleaning
	A4	Fingernail and toenail care
	A5	Assistance with sponge bath and showering
	A6	Assistance with eating/drinking and guidance
	A7	Assistance with dressing and guidance
	A8	Assistance with excretion and guidance
	A9	Making up bed units
	A10	Assistance with in‐bed position changes
	A11	Assistance with mobility using assistive devices
	A12	Safety protection and guidance
	A13	Pressure injury prevention and guidance

Medical Care and Rehabilitation	B1	Vital signs monitoring
	B2	Cryotherapy and thermotherapy
	B3	Oxygen therapy
	B4	Non‐invasive ventilation
	B5	Nebulized inhalation
	B6	Sputum suction
	B7	Mechanical‐assisted sputum expectoration
	B8	Tracheotomy care
	B9	Nasal feeding
	B10	Oral medication administration and medication guidance
	B11	Specimen collection
	B12	Urinary catheterization
	B13	Indwelling catheter care
	B14	Enema and anal tube
	B15	Incontinence care
	B16	Ostomy care
	B17	Blood glucose testing
	B18	Insulin injection
	B19	Intravenous indwelling needle care
	B20	Medication administration guidance and care
	B21	Pressure injury/wound care
	B22	Physical health assessment and post‐assessment education
	B23	Physical function rehabilitation training
	B24	Respiratory function training
	B25	Incontinence function training
	B26	Cognitive training
	B27	Language training

Psychological Care	C1	Psychological assessment
	C2	Psychological support
	C3	Psychological communication and counseling

Traditional Chinese Medicine Nursing	D1	Scraping (Gua Sha)
	D2	Cupping (Ba Guan)
	D3	Moxibustion (Ai Jiu)
	D4	Traditional Chinese medicine soaking and cleansing
	D5	Acupoint application
	D6	External application of traditional Chinese medicine
	D7	TCM medication administration care
	D8	TCM emotional care
	D9	TCM dietary care

**TABLE 3:** Kano model demand attribute classification matrix.

Positive question	Negative question				
	I like it	I expect it	Neutral	I can tolerate it	I dislike it
I like it	Q	A	A	A	O
I expect it	R	I	I	I	M
Neutral	R	I	I	I	M
I can tolerate it	R	I	I	I	M
I dislike it	R	R	R	R	Q

*Note:* M = Must‐be; O = One‐dimensional; A = Attractive; I = Indifferent; Q = Questionable; R = Reverse.

##### 2.3.3.3. Reliability and Validity

A pre‐test was conducted with 80 eligible disabled older adult participants to evaluate the psychometric properties of the questionnaire. Reliability analysis showed excellent internal consistency, with Cronbach’s *α* coefficients of 0.963 for the positive questions and 0.969 for the negative questions. The Cronbach’s *α* for individual dimensions ranged from 0.938 to 0.968. Validity was assessed using the KMO measure and Bartlett’s test of sphericity. The KMO values were 0.920 for positive questions and 0.933 for negative questions, and Bartlett’s tests were significant (both *p* < 0.001), indicating good construct validity.

### 2.4. Data Collection

The investigation team consisted of two geriatric nursing experts and five nursing graduate students who underwent standardized training on the study protocol and questionnaire administration. Data collection was conducted through face‐to‐face interviews. Before the survey, researchers explained the study’s purpose and obtained written informed consent from each participant. To ensure accuracy, investigators assisted participants in completing the questionnaires and promptly reviewed each questionnaire on‐site to address any missing or ambiguous responses. Each interview lasted approximately 30 min. A small gift was provided to participants as a token of appreciation.

### 2.5. Quality Control

To ensure data quality, several measures were implemented. During the study design phase, the inclusion and exclusion criteria were clearly defined, and a pilot test was conducted to refine the questionnaire. During formal data collection, investigators were trained to use neutral language and avoid leading questions. All completed questionnaires were double‐checked by two researchers for completeness and logical consistency. Data entry was performed independently by two researchers using SPSS 26.0, with discrepancies resolved by referring back to the original questionnaires.

### 2.6. Ethical Approval

This study was approved by the Medical Ethics Committee of a participating tertiary hospital (approval no.: 2022KL‐033). Written informed consent was obtained from all participants prior to data collection.

### 2.7. Statistical Analysis

Data were analyzed using SPSS 26.0 (IBM Corp., Armonk, NY, USA), Microsoft Excel 2021, and Python 3.8. Descriptive statistics were used to summarize participants′ sociodemographic characteristics and disability levels, with categorical variables expressed as frequencies and percentages.

The Kano model was used to classify the attributes of LTC service demands. According to the standard Kano methodology [27, 28], each service item was assigned to one of five categories (attractive, one‐dimensional, must‐be, indifferent, and reverse) based on the highest frequency in the classification matrix. To refine this classification, the better–worse coefficient was calculated using the following formulas [11]:

(1) SI=A+OA+O+M+I,DSI=−O+MA+O+M+I.

A quadrant chart was constructed with the absolute value of Dissatisfaction Index (DSI) on the *x*‐axis and Satisfaction Index (SI) on the *y*‐axis to visually represent the distribution of service attributes. Following the common practice in Kano‐based analyses, the mean values of SI and |DSI| were used as the quadrant boundaries, a common practice in Kano‐based analyses to ensure balanced classification. Services in the first quadrant (high SI, high |DSI|) were classified as one‐dimensional (O) attributes; those in the second quadrant (high SI, low |DSI|) as attractive (A) attributes; those in the third quadrant (low SI, low |DSI|) as indifferent (I) attributes; and those in the fourth quadrant (low SI, high |DSI|) as must‐be (M) attributes.

To establish a precise supply priority order, we applied entropy‐weighted TOPSIS [29]. The better (SI) and worse (|DSI|) coefficients were used as evaluation indicators, with weights objectively determined via the entropy method [30]. The relative closeness (*C*_*i*_) to the ideal solution was calculated for each service item; higher *C*_*i*_ values indicate higher supply priority. Importantly, because TOPSIS prioritizes continuous *C*_*i*_ values rather than discrete Kano labels alone, it enables fine‐grained urgency differentiation even among services within the same Kano category.

## 3. Results

### 3.1. Participant Characteristics

A total of 585 disabled older adults completed the survey, with a valid response rate of 95.9%. The mean age of participants was 76.4 ± 8.2 years, and the majority were female (56.6%). Regarding disability level, 306 participants (52.3%) were classified as mildly disabled, 144 (24.6%) as moderately disabled, 88 (15.0%) as severely disabled, and 47 (8.0%) as completely disabled. In terms of care setting, 298 participants (50.9%) resided in institutional care facilities, while 287 (49.1%) were community‐dwelling older adults receiving home‐based care. Detailed participant characteristics are presented in Table 4.

**TABLE 4:** Sociodemographic and health‐related characteristics of participants (*N* = 585).

Characteristic	Category	*n*	%
Gender	Male	254	43.4
	Female	331	56.6

Age (years)	60–69	116	19.8
	70–79	199	34.0
	80–89	184	31.5
	≥ 90	86	14.7

Educational level	Illiterate	72	12.3
	Primary school	176	30.1
	Junior high school	187	32.0
	Senior high school or technical secondary school	95	16.2
	College or above	55	9.4

Marital status	Married	280	47.9
	Unmarried/divorced	73	12.5
	Widowed	232	39.7

Living arrangement	Living with children or relatives	109	18.6
	Living alone	273	46.7
	Living with spouse	203	34.7

Number of children	0	11	1.9
	1	196	33.5
	2	231	39.5
	≥ 3	147	25.1

Primary source of income	Pension	452	77.3
	Savings	84	14.4
	Financial support from children/relatives	39	6.7
	Government subsidy	10	1.7

Monthly household income per capita (RMB)	< 2000	14	2.4
	2000–3999	176	30.1
	4000–5999	187	32.0
	6000–9999	118	20.2
	≥ 10,000	90	15.4

Type of medical insurance	Urban employee basic medical insurance	280	47.9
	Urban resident basic medical insurance	102	17.4
	New rural cooperative medical scheme	193	32.9
	None	10	1.7

Primary caregiver	None	47	8.0
	Spouse	187	32.0
	Children	101	17.3
	Paid caregiver	241	41.2
	Others	9	1.5

Number of chronic diseases	0	32	5.5
	1	130	22.2
	2	210	35.9
	≥ 3	213	36.4

Number of medications taken	0	28	4.8
	1	65	11.1
	2	149	25.5
	≥ 3	343	58.6

Disability level	Mild disability	306	52.3
	Moderate disability	144	24.6
	Severe disability	88	15.0
	Complete disability	47	8.1

Care setting	Nursing institution	298	50.9
	Community‐dwelling	287	49.1

### 3.2. Better–Worse Coefficient Analysis and Quadrant Classification

**FIGURE 1:**
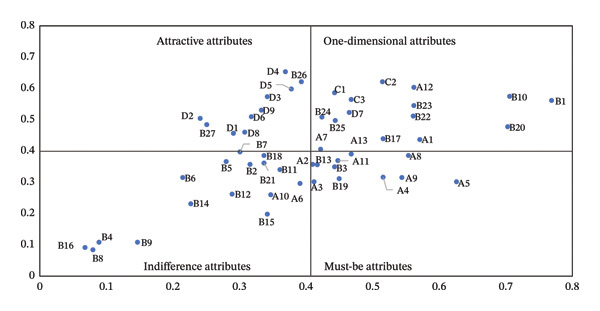
Better–worse coefficient quadrant plot of long‐term care service demand attributes among disabled older adults (*N* = 585).

The better (SI) and worse (DSI) coefficients were calculated for all 52 service items. SI values ranged from 0.084 to 0.653 (mean = 0.407), while |DSI| values ranged from 0.068 to 0.769 (mean = 0.399). The mean SI and mean |DSI| were used as the quadrant boundaries for the two‐dimensional quadrant chart (Figure 1).

As shown in Table 5 and Figure 1, based on the final classification using the better–worse coefficients, 12 service items were categorized as must‐be attributes, all located in the fourth quadrant (low SI, high |DSI|). These included oral care (A2, SI = 0.357, |DSI| = 0.410), bathing assistance (A5, SI = 0.301, |DSI| = 0.626), excretion care and guidance (A8, SI = 0.385, |DSI| = 0.554), bed unit management (A9, SI = 0.315, |DSI| = 0.544), and oxygen therapy (B3, SI = 0.349, |DSI| = 0.443), among others (see Table 5 for the full list). These services are essential for maintaining basic quality of life; their absence leads to significant dissatisfaction.

**TABLE 5:** Better–worse coefficients and final demand attributes of long‐term care service items (*N* = 585).

Code	Service item	SI	|DSI|	Final attribute
*Daily care*				
A1	Head and facial cleansing and grooming	0.436	0.571	O
A2	Oral care	0.357	0.410	M
A3	Hand and foot cleaning	0.301	0.412	M
A4	Fingernail and toenail care	0.316	0.516	M
A5	Assistance with bathing or sponging	0.301	0.626	M
A6	Assistance with eating/drinking and guidance	0.296	0.391	I
A7	Assistance with dressing and guidance	0.405	0.422	M
A8	Excretion care and guidance	0.385	0.554	M
A9	Bed unit management	0.315	0.544	M
A10	Assistance with in‐bed positioning and transfer	0.260	0.347	I
A11	Assistance with assistive device use for mobility	0.369	0.448	M
A12	Safety protection and guidance	0.603	0.562	O
A13	Pressure injury prevention and guidance	0.390	0.468	M

*Medical and rehabilitation care*				
B1	Vital signs monitoring	0.561	0.769	O
B2	Cold and heat therapy	0.357	0.316	I
B3	Oxygen therapy	0.349	0.443	M
B4	Non‐invasive ventilation	0.108	0.089	I
B5	Nebulized inhalation	0.366	0.280	I
B6	Sputum suctioning	0.315	0.215	I
B7	Mechanically assisted sputum expectoration	0.397	0.301	I
B8	Tracheotomy care	0.084	0.080	I
B9	Nasogastric feeding	0.108	0.147	I
B10	Oral medication administration and guidance	0.574	0.706	O
B11	Specimen collection	0.340	0.361	I
B12	Urinary catheterization	0.262	0.289	I
B13	Indwelling catheter care	0.356	0.417	M
B14	Enema and rectal tube	0.231	0.227	I
B15	Incontinence care	0.198	0.342	I
B16	Ostomy care	0.091	0.068	I
B17	Blood glucose monitoring	0.439	0.516	O
B18	Insulin injection	0.385	0.337	I
B19	Intravenous indwelling needle care	0.311	0.450	M
B20	Medication administration guidance and care	0.477	0.703	O
B21	Pressure injury/wound care	0.361	0.337	I
B22	Health assessment and post‐assessment education	0.511	0.561	O
B23	Physical function rehabilitation training	0.545	0.562	O
B24	Respiratory function training	0.508	0.424	O
B25	Incontinence function training	0.497	0.444	O
B26	Cognitive training	0.621	0.393	A
B27	Language training	0.484	0.251	A

*Psychological care*				
C1	Psychological assessment	0.586	0.443	O
C2	Psychological support	0.621	0.515	O
C3	Psychological communication and counseling	0.564	0.468	O

*Traditional Chinese medicine (TCM) care*				
D1	Scraping therapy (Gua sha)	0.456	0.291	A
D2	Cupping therapy	0.504	0.241	A
D3	Moxibustion	0.573	0.342	A
D4	TCM herbal soaking	0.653	0.369	A
D5	Acupoint application	0.598	0.378	A
D6	TCM external application	0.509	0.318	A
D7	TCM administration care	0.523	0.465	O
D8	TCM emotional nursing	0.460	0.308	A
D9	TCM dietary care	0.530	0.333	A

Fourteen service items were identified as one‐dimensional attributes, all situated in the first quadrant (high SI, high |DSI|). Representative services included vital signs monitoring (B1, SI = 0.561, |DSI| = 0.769), oral medication administration and guidance (B10, SI = 0.574, |DSI| = 0.706), and psychological support (C2, SI = 0.621, |DSI| = 0.515). These services strongly influence satisfaction when provided and cause dissatisfaction when absent.

Ten service items were classified as attractive attributes, distributed in the second quadrant (high SI, low |DSI|). These were predominantly TCM services, including TCM herbal soaking (D4, SI = 0.653, |DSI| = 0.369), moxibustion (D3, SI = 0.573, |DSI| = 0.342), and cupping (D2, SI = 0.504, |DSI| = 0.241), as well as cognitive training (B26, SI = 0.621, |DSI| = 0.393). These services substantially enhance satisfaction when provided but do not cause dissatisfaction when absent.

Sixteen service items were categorized as indifferent attributes, located in the third quadrant (low SI, low |DSI|). These included specialized nursing procedures such as tracheotomy care (B8, SI = 0.084, |DSI| = 0.080), nasogastric feeding (B9, SI = 0.108, |DSI| = 0.147), and ostomy care (B16, SI = 0.091, |DSI| = 0.068). These services had minimal impact on satisfaction regardless of whether they were provided.

### 3.3. Supply Priority Ranking Based on TOPSIS Method

**FIGURE 2:**
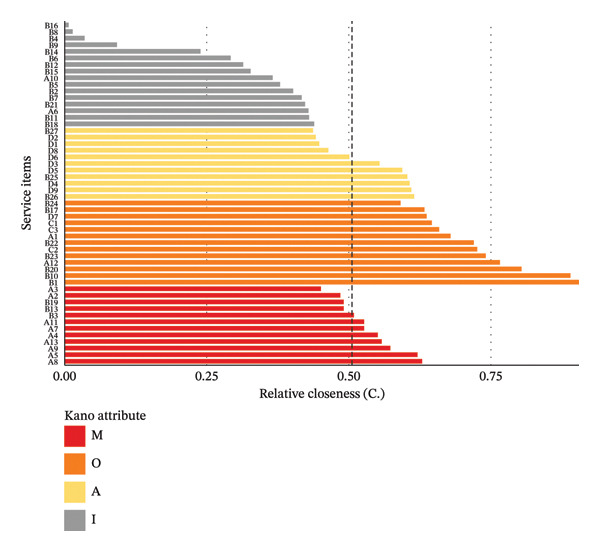
Tiered supply priority classification of long‐term care service items based on the TOPSIS method.

Although the Kano model effectively classifies service attributes into must‐be (M), one‐dimensional (O), attractive (A), and indifferent (I) categories, it does not allow for fine‐grained prioritization within the same attribute category. To address this limitation and establish a precise supply priority order for the 52 LTC service items, we applied the entropy‐weighted TOPSIS method. The better (SI) and worse (|DSI|) coefficients derived from the Kano analysis were used as evaluation indicators, and their weights were objectively determined via the entropy method. The relative closeness (*C*_*i*_) to the ideal solution was calculated for each service item; a higher *C*_*i*_ value indicates a greater urgency for supply. In TOPSIS, *D* ^+^ represents the distance between each service item and the positive ideal solution (the best possible performance across all criteria), whereas *D* ^−^ represents the distance from the negative ideal solution (the worst possible performance). The relative closeness coefficient *C*_*i*_ is then calculated as *C*_*i*_ = *D* ^−^(*D* ^+^ + *D* ^−^), yielding a value between 0 and 1 that reflects how close a service item is to the ideal state. A higher *C*_*i*_ value indicates that a service has a stronger combined effect on satisfaction enhancement and dissatisfaction reduction, and therefore should receive higher supply priority. It should be noted that the final priority order follows a two‐step logic: first, services are prioritized by Kano category following the strategic hierarchy of Must‐be > One‐dimensional > Attractive > Indifferent; second, within each Kano category, services are further prioritized by their *C*_*i*_ values. This ensures that essential services are guaranteed first, while satisfaction‐enhancing services are introduced in subsequent phases. Table 6 presents the positive ideal solution distance (*D* ^+^), negative ideal solution distance (*D* ^−^), and relative closeness (*C*_*i*_) for all 52 items. Based on these *C*_*i*_ values, the service items were prioritized within each Kano category, with the resulting order presented in Figure 2.

**TABLE 6:** Relative closeness (*C* *i*) of long‐term care service items based on entropy‐weighted TOPSIS (*N* = 585).

Code	Service item	*D* ^+^	*D* ^−^	*C* *i*
B1	Vital signs monitoring	0.020	0.196	0.905
B10	Oral medication administration and guidance	0.023	0.185	0.890
B20	Medication administration guidance and care	0.042	0.173	0.804
A12	Safety protection and guidance	0.050	0.164	0.766
B23	Physical function rehabilitation training	0.054	0.155	0.741
C2	Psychological support	0.060	0.159	0.726
B22	Health assessment and post‐assessment education	0.058	0.150	0.720
A1	Head and facial cleansing and grooming	0.067	0.142	0.679
C3	Psychological communication and counseling	0.073	0.142	0.659
C1	Psychological assessment	0.078	0.142	0.646
D7	Traditional Chinese medicine administration care	0.077	0.135	0.637
B17	Blood glucose monitoring	0.076	0.132	0.633
A8	Excretion care and guidance	0.078	0.132	0.629
A5	Assistance with bathing or sponging	0.085	0.140	0.621
B26	Cognitive training	0.089	0.142	0.615
D9	Traditional Chinese medicine dietary care	0.152	0.238	0.610
D4	Traditional Chinese medicine herbal soaking	0.094	0.145	0.607
B25	Incontinence function training	0.084	0.127	0.603
D5	Acupoint application	0.093	0.135	0.594
B24	Respiratory function training	0.087	0.126	0.591
A9	Bed unit management	0.092	0.123	0.573
D3	Moxibustion	0.102	0.126	0.554
A13	Pressure injury prevention and guidance	0.092	0.116	0.558
A4	Fingernail and toenail care	0.096	0.117	0.551
A7	Assistance with dressing and guidance	0.098	0.110	0.527
A11	Assistance with assistive device use for mobility	0.098	0.109	0.527
B3	Oxygen therapy	0.102	0.106	0.509
D6	Traditional Chinese medicine external application	0.111	0.111	0.501
B13	Indwelling catheter care	0.106	0.102	0.491
B19	Intravenous indwelling needle care	0.107	0.103	0.491
A2	Oral care	0.107	0.101	0.485
D8	Traditional Chinese medicine emotional nursing	0.116	0.101	0.464
D1	Scraping therapy (Gua sha)	0.121	0.098	0.448
B18	Insulin injection	0.118	0.092	0.439
B27	Language training	0.127	0.099	0.437
B11	Specimen collection	0.118	0.089	0.430
A6	Assistance with eating/drinking and guidance	0.119	0.089	0.429
B21	Pressure injury/wound care	0.120	0.088	0.423
B7	Mechanical assisted sputum expectoration	0.124	0.089	0.417
B2	Cold and heat therapy	0.125	0.084	0.402
B5	Nebulized inhalation	0.131	0.080	0.379
A10	Assistance with in‐bed positioning and transfer	0.132	0.076	0.366
B15	Incontinence care	0.142	0.069	0.327
B12	Urinary catheterization	0.142	0.065	0.314
B6	Sputum suctioning	0.150	0.062	0.292
B14	Enema and rectal tube	0.158	0.050	0.239
B9	Nasogastric feeding	0.190	0.019	0.092
B4	Non‐invasive ventilation	0.200	0.007	0.035
B8	Tracheotomy care	0.205	0.003	0.014
B16	Ostomy care	0.207	0.002	0.007

*Note:* *D* ^+^ = distance to positive ideal solution; *D* ^−^ = distance to negative ideal solution; *C* *i* = relative closeness to the ideal solution, ranging from 0 to 1.

## 4. Discussion

This study employed an integrated Kano–TOPSIS framework to characterize the hierarchical structure of LTC demands and the corresponding supply priorities among 585 disabled older adult individuals in Chengdu, China. The Kano model classified 52 service items into must‐be (12), one‐dimensional (14), attractive (10), and indifferent (16) attributes, revealing a clear hierarchy progressing from foundational physiological needs to higher order psychological expectations, consistent with Maslow’s hierarchy of needs [31]. A notable pattern of “expectations outweighing basic dependence” emerged, with most items exhibiting a higher SI than |DSI|. By integrating entropy‐weighted TOPSIS within each Kano category, we established fine‐grained supply priorities based on relative closeness (*C*_*i*_) values, enabling resource allocation that accounts for both strategic category and within‐category urgency. TCM nursing services were predominantly classified as attractive or one‐dimensional attributes, highlighting their potential as value‐added components in culturally appropriate contexts.

The hierarchical structure identified by the Kano model underscores the need to differentiate service strategies. Must‐be attributes—bathing assistance (A5) and excretion care (A8), for instance—constitute non‐negotiable basics; their absence provokes sharp dissatisfaction, whereas their mere presence does little to enhance satisfaction, and thus these services should be guaranteed as a basic package. One‐dimensional attributes—including vital signs monitoring (B1), medication administration (B10), and psychological support (C2)—display a linear relationship with service provision; improvements translate directly into higher satisfaction, positioning them as key targets for quality monitoring and performance‐based reimbursement. This aligns with prior research emphasizing that effective needs assessment should inform priority setting [19] and that when basic needs are met, older adults increasingly value services that foster autonomy and meaningful engagement [32]. Attractive attributes offer opportunities to exceed expectations without creating deprivation when unavailable [22]—in this study, such services were primarily TCM items like herbal soaking (D4) and moxibustion (D3), along with cognitive training (B26). Indifferent attributes—tracheotomy care (B8) and ostomy care (B16), for example—have minimal impact on overall satisfaction and thus merit cautious prioritization in resource‐limited contexts, although services with higher *C*_*i*_ values within this category, such as indwelling catheter care (B13), deserve consideration for subpopulations with specific needs. As caregivers, we should distinguish non‐negotiable, satisfaction‐driven, and value‐added services, thereby allocating limited resources more effectively and responding to the true priorities of disabled older adults.

The integration of TOPSIS within each Kano category addressed the common assumption of homogeneity within attribute categories and provided actionable supply sequences. Among must‐be attributes, *C*_*i*_ values varied substantially: excretion care (A8, *C*_*i*_ = 0.629) and bathing assistance (A5, *C*_*i*_ = 0.621) showed significantly higher urgency than oral care (A2, *C*_*i*_ = 0.485), indicating that even foundational services should be prioritized based on their impact on dignity, hygiene, and safety. Within one‐dimensional attributes, vital signs monitoring (B1, *C*_*i*_ = 0.905), oral medication administration (B10, *C*_*i*_ = 0.890), and safety protection (A12, *C*_*i*_ = 0.766) ranked highest, yielding the most immediate satisfaction gains per unit of resource invested. These essential medical services are critical to health maintenance and safety, echoing previous findings that disabled older adults have fundamental needs for routine health monitoring and medication support [33, 34]. For attractive attributes, cognitive training (B26, *C*_*i*_ = 0.615) demonstrated the highest priority, addressing cognitive decline—a condition closely linked to daily functioning and social participation [35]. This two‐step approach—strategic classification by Kano followed by within‐category prioritization by *C*_*i*_—enables evidence‐based, phased implementation that maximizes satisfaction under resource constraints. Based on this two‐step approach, nursing managers can obtain a practical guide for stepwise resource allocation, ensuring that the most critical services are delivered first while still attending to the nuanced priorities of disabled older adults.

A distinctive finding with broad implications is the “expectations outweighing basic dependence” pattern, wherein most services had higher SI than |DSI|. The highest SI values were observed for TCM herbal soaking (D4, SI = 0.653), cognitive training (B26, SI = 0.621), and psychological support (C2, SI = 0.621), indicating their greatest potential to improve satisfaction. These findings align with prior evidence showing that TCM interventions may alleviate pain, improve physical function, and delay functional decline [36–38], while cognitive training and psychological support address the often‐overlooked cognitive and emotional needs of disabled older adults, which are frequently exacerbated by loss of independence and social isolation [39, 40]. This pattern signals a fundamental shift in how disabled older adults perceive care: while dependence on basic services remains, expectations increasingly extend toward services that enhance quality of life, autonomy, and emotional well‐being. This trend reflects China’s rapid economic development, rising educational levels, and growing exposure to person‐centered care concepts. Consequently, merely preventing dissatisfaction is no longer sufficient; LTC systems must proactively invest in services that generate satisfaction gains, particularly those with high SI such as cognitive training, psychological support, and TCM wellness services. Within this context, TCM nursing services—largely attractive attributes—represent strategic opportunities for service differentiation. In resource‐rich settings, they can yield high satisfaction returns; in resource‐constrained environments, they should be considered optional enhancements after must‐be and high‐priority one‐dimensional services are secured. However, the evidence base for TCM nursing in geriatric care remains emerging [41], and workforce capacity gaps necessitate integration of TCM competencies into nursing education [42]. Together, these findings call for shifting focus from preventing dissatisfaction to actively cultivating satisfaction—by investing in high‐value services and building the evidence and competencies to deliver them.

These findings have important implications for LTC policy and practice. First, must‐be services, such as bathing assistance, excretion care, oxygen therapy, pressure injury prevention, and safety‐related support, should be guaranteed as a basic service package to protect dignity, safety, and essential daily functioning. Second, one‐dimensional services, including vital signs monitoring, medication administration, rehabilitation training, psychological support, and health assessment, should be incorporated into quality monitoring, performance evaluation, and LTC insurance payment standards, because improvement in these services directly increases satisfaction. Third, attractive services, particularly cognitive training and TCM nursing services, can be developed as value‐added service modules to enhance satisfaction, cultural responsiveness, and service differentiation. In resource‐limited settings, policymakers and nursing managers may adopt a phased allocation strategy: first ensuring must‐be services, then strengthening high‐priority one‐dimensional services, and finally expanding attractive services according to local resource capacity. Practical implementation should also consider service cost, workforce availability, institutional capacity, insurance reimbursement, and policy feasibility.

Several limitations should be acknowledged. First, the cross‐sectional design captures needs at a single time point and cannot reflect how preferences evolve as health status changes or how needs structures may differ across disability levels. Longitudinal studies are therefore needed to examine these dynamic changes. Second, the study was conducted in Chengdu, which may limit the generalizability of the findings to regions with different cultural backgrounds or LTC infrastructure. Third, the use of convenience sampling may introduce selection bias. Our sample consisted primarily of individuals with mild to moderate disability, while those with severe or complete disability were underrepresented. Future studies with purposive oversampling and larger, more balanced samples are needed to enable robust subgroup analyses and further validate our findings. In addition, future research could enhance the practical applicability of these findings by incorporating cost, workforce, and policy indicators into the TOPSIS framework. Despite these limitations, the integrated Kano–TOPSIS framework offers a rigorous, user‐centered approach to needs assessment and resource allocation that can inform policy and practice in the development of responsive LTC systems.

## 5. Conclusions

This study employed an integrated Kano–TOPSIS framework to assess the hierarchical structure and supply priorities of LTC needs among disabled older adults in Chengdu. Care demands followed a clear hierarchy: must‐be attributes formed the non‐negotiable foundation; one‐dimensional attributes directly drove satisfaction; attractive attributes offered opportunities to exceed expectations; and indifferent attributes warranted cautious resource allocation. TOPSIS enabled fine‐grained prioritization within each category, providing actionable guidance for phased service implementation. TCM nursing services emerged predominantly as attractive or one‐dimensional attributes, highlighting their value in culturally responsive care systems. The observed pattern of expectations exceeding dependence reflects a shift in care priorities toward quality of life and autonomy. Overall, the integrated Kano–TOPSIS framework provides a rigorous, user‐centered approach to needs assessment and resource allocation, with important implications for developing responsive LTC systems.

## Funding

This research was supported by the Chengdu Philosophy and Social Science Planning Project (Grant No. 2024BS015).

## Conflicts of Interest

The authors declare no conflicts of interest.

## Data Availability

The datasets generated and/or analyzed during the current study are not publicly available due to privacy and ethical restrictions but are available from the corresponding author on reasonable request.
